# Synergistic Effect of Family History of Diabetes and Dietary Habits on the Risk of Type 2 Diabetes in Central China

**DOI:** 10.1155/2017/9707284

**Published:** 2017-04-13

**Authors:** Yanyan Zhao, Chunhua Song, Xiaokun Ma, Xiaojun Ma, Qingzhu Wang, Hongfei Ji, Feng Guo, Guijun Qin

**Affiliations:** ^1^Division of Endocrinology, Department of Internal Medicine, The First Affiliated Hospital, Zhengzhou University, Zhengzhou 450052, China; ^2^Department of Epidemiology and Biostatistics, College of Public Health, Zhengzhou University, Zhengzhou 450052, China

## Abstract

*Background*. Family history of diabetes (FHD) and lifestyle are associated with type 2 diabetes (T2DM), but little is known about the FHD diet interactions. We aimed to analyze the interactions of FHD and lifestyle factors in Chinese T2DM onset. *Methods*. This was a cross-sectional survey in central urban China (*n* = 1234 patients with T2DM and *n* = 8615 non-T2DM subjects). The biological interactions, defined by Rothman interactions, between FHD and each dietary factor were analyzed by using the synergy index (*S*) scores. *Results*. After adjustment for age, gender, BMI, and WHR, a uniparental FHD (OR = 2.84, 95% CI: 2.36–3.42, *P* < 0.001), a paternal history of FHD (OR = 2.53, 95% CI: 1.91–3.35, *P* < 0.001), a maternal history of FHD (OR = 3.27, 95% CI: 2.67–4.02, *P* < 0.001), a biparental history of FHD (OR = 5.26, 95% CI: 2.98–9.31, *P* < 0.001), and a FHD, irrespective of the parent (OR = 3.59, 95% CI: 3.08–4.17, *P* < 0.001), were associated with T2DM onset. There were significant interactions between FHD and consuming <15 g/d of potatoes (*S* = 1.54, 95% CI: 1.12–2.12), <8 g/d of poultry (*S* = 1.51, 95% CI: 1.04–2.17), <85 g/d of fresh fruits (*S* = 2.17, 95% CI: 1.63–2.88), and no freshly squeezed juice (*S* = 2.25, 95% CI: 1.46–3.49). *Conclusions*. Risk of T2DM was synergistically affected by FHD and dietary habits. Nutrition educational intervention may decrease the prevalence of T2DM in the Chinese with FHD.

## 1. Introduction

Diabetes is now seen as a global epidemic [1]. According to the statistics published by the World Health Organization (WHO) in 2011, the prevalence of diabetes around the world has reached 366 million and most of these patients have type 2 diabetes mellitus (T2DM) [2]. The prevalence of diabetes has increased significantly in recent decades and is now reaching epidemic proportions in China [3]. Compared with 1980, the prevalence of T2DM has increased by a factor of 3 in 1994 and has approximately doubled from 1994 to 2001 [3].

Dietary habits are well known to influence the risk of T2DM. A Western pattern diet (high consumption of red meat, processed meat, refined grains, French fries, high-fat dairy products, sweets and desserts, high-sugar drinks, and eggs) has been associated with an increased risk of T2DM in both men and women [4, 5]. The Chinese have a unique dietary pattern that might have protected them from T2DM in the past, but westernization of the dietary habits in China during the recent decades may participate in the obesity and T2DM epidemics observed in China [6–8]. Nevertheless, the exact relationship between T2DM and dietary habits in China is currently poorly understood.

Recent studies reported that family history of diabetes (FHD) is associated with an increased prevalence of T2DM [9–11]. Those with a parental history of diabetes are more susceptible to suffer from T2DM compared with those without parental history [9–11]. It is likely that this elevated risk of T2DM is mediated, in part, by both genetic and shared environmental components among family members [12], but whether the FHD has the same impact on the risk of T2DM is unclear. Similarly, although some previous studies revealed that anthropometric and lifestyle-related risk factors such as body mass index (BMI), waist circumference, and physical inactivity are major risk factors for T2DM [13–15] and that the aggregation of such traits among families may account for a portion of the excess risk attributable to FHD [16], the precise factors accounting for this increase in risk are poorly understood. Moreover, the current reports about the interaction of FHD with lifestyle risk factors are few. After a first-degree relative experiences T2DM, it might be expected that other family members would take this as a warning which might lead to changes in risk factor exposure. This might be reflected in differences in risk factor exposure and odds ratios (ORs) between individuals with and without FHD.

To answer these questions, the present study was carried out using data from Henan province's study sites of the Chinese Center for Disease Control and Prevention's (CDC's) National Disease Surveillance Point System. Differences in lifestyle were compared to FHD status in patients with T2DM and non-T2DM subjects for possible relationships. The interactions between them were also analyzed.

## 2. Methods

### 2.1. Study Design and Subjects

The present work was one part of the baseline survey from REACTION study investigating the association of diabetes and cancer, which was conducted among 259,657 adults, aged 40 years and older in 25 communities across mainland China, from 2011 to 2012 [17, 18]. All subjects' data were drawn from the REACTION study. This survey was conducted in four communities in Zhengzhou city, Henan province, from July 2010 to August 2010. In this previous cross-sectional study, a complex, multistage, probability sampling design was used to select participants. This process aimed to select a study sample that was representative of civilian, noninstitutionalized Chinese adults at each site. One individual of ≥18 years of age was randomly selected from each household. If the selected individuals refused or were unavailable, a similar and previously unselected replacement household was selected in the same neighborhood.

The original study was approved by Ruijin Hospital Ethics Committee. Written informed consent was obtained from all study participants. The present study was approved by the same ethics committee, but the need for individual consent was waived because of the retrospective nature of the study.

### 2.2. Data Collection

A standard questionnaire was administered by trained staff to obtain information on demographic characteristics, personal and family medical history, and lifestyle risk factors [19]. A pilot study was first conducted on a small group of district residents to test the validity of the questionnaire. “Current smoking” was defined as having smoked 100 cigarettes in one's lifetime. Previous smoking was defined as having stopped smoking for at least 1 year. Similarly, “Current drinking” was defined as the consumption of at least 30 g of alcohol per week for 1 year or more. Consumption of milk, eggs, meat (chicken, beef, and pork), raw vegetables, fruits, and other dietary items was divided into two or three categories according to intake frequency. Information was obtained on the amount and type of alcohol that was consumed during the previous year.

Bodyweight and height were measured according to a standard protocol, and BMI was calculated as weight in kilograms divided by height in meters squared. Waist circumference was measured on standing participants midway between the lower edge of the costal arch and the upper edge of the iliac crest.

In this study, FHD was defined as positive if the subject had at least one parent or sibling or children who had been diagnosed with T2DM.

### 2.3. Statistical Analyses

The Pearson chi-square test was used to assess the differences in the frequency distribution of the categorical variables between T2DM and non-T2DM. The Mann–Whitney *U* test was used to assess the differences in non-normally distributed continuous variables between T2DM and non-T2DM. Multivariate logistic regression was performed to obtain OR estimates and their 95% confidence intervals (95% CI) for lifestyle factors on T2DM onset. The estimates were adjusted for age, sex, BMI, waist-to-hip ratio (WHR), and FHD. The biological interactions, defined by Rothman interactions [20], between FHD and each dietary factor were analyzed by using the synergy index (*S*) scores [19]. An *S* score of >1.0 indicates positive interaction and an *S* score of below <1.0 indicates an antagonistic effect [21]. All of the statistical tests were performed using SPSS 16.0 (IBM, Armonk, NY, USA). Two-sided *P* values <0.05 were considered statistically significant.

## 3. Results

### 3.1. Characteristics of the Patients

The present study included 9849 representative urban residents, including 1234 hospital-diagnosed patients with T2DM and 8615 non-T2DM individual. Compared with non-T2DM individuals, patients with T2DM showed a higher proportion of males (38.7% versus 31.4%, *P* < 0.001), older age (median, 63 versus 58 years, *P* < 0.001), higher BMI (median, 26.2 versus 25.7 kg/m^2^, *P* < 0.001), and higher WHR (0.918 versus 0.896, *P* < 0.001) (Table 1).

Among the patients with T2DM, 332 (26.9%) were FHD+ compared with 1025 (11.9%) among non-T2DM individuals (*P* < 0.001). Compared with non-T2DM individuals, patients with T2DM showed a higher proportion of patients with a history of smoking ≥ 7 cigarettes/week (10.9% versus 7.3%, *P* < 0.001), a lower consumption of potatoes (*P* < 0.001), pork (*P* < 0.001), fresh fruits (*P* < 0.001), and freshly squeezed fruits (*P* < 0.001), a higher consumption of poultry (*P* = 0.002), and a higher level of physical activity (*P* < 0.001) (Table 2).

### 3.2. Association between FHD and T2DM

After adjustment for age, gender, BMI, and WHR, a uniparental FHD (OR = 2.84, 95% CI: 2.36–3.42, *P* < 0.001), a paternal history of FHD (OR = 2.53, 95% CI: 1.91–3.35, *P* < 0.001), a maternal history of FHD (OR = 3.27, 95% CI: 2.67–4.02, *P* < 0.001), a biparental history of FHD (OR = 5.26, 95% CI: 2.98–9.31, *P* < 0.001), and a FHD, irrespective of the parent (OR = 3.59, 95% CI: 3.08–4.17, *P* < 0.001), were associated with T2DM onset (Table 3).

### 3.3. Effect of Lifestyle Factors on T2DM Onset

After adjustment for age, gender, BMI, and WHR, the consumption of potatoes (<15 g/d, OR = 1.49, 95% CI: 1.30–1.71, *P* < 0.001), beef and mutton (<4 g/d, OR = 0.78, 95% CI: 0.68–0.91, *P* < 0.001), fresh fruits (<85 g/d, OR = 2.34, 95% CI: 2.04–2.68, *P* < 0.001), freshly squeezed juice (no, OR = 2.23, 95% CI: 1.86–2.68, *P* < 0.001), and soy products (≥30 g/d, OR = 1.19, 95% CI: 1.04–1.37, *P* = 0.01), and days of walking/week (>3, OR = 1.29, 95% CI: 1.11–1.49, *P* = 0.001) were associated with T2DM onset (Table 4).

### 3.4. Interactions between Lifestyle Habits and FHD

As shown in Table 5, there were significant interactions between FHD and consuming < 15 g/d of potatoes (*S* = 1.54, 95% CI: 1.12–2.12), <8 g/d of poultry (*S* = 1.51, 95% CI: 1.04–2.17), <85 g/d of fresh fruits (*S* = 2.17, 95% CI: 1.63–2.88), and no freshly squeezed juice (*S* = 2.25, 95% CI: 1.46–3.49).

## 4. Discussion

T2DM is a major public health problem in China [22, 23]. Fortunately, there are preventive measures, and persons at risk can be readily identified using a few common risk factors [24]. In this study, we conducted a study to identify lifestyle risk factors of T2DM and their interactions with FHD in a Chinese urban population.

After adjustment for age, gender, BMI, and WHR, a uniparental FHD, a paternal history of FHD, a maternal history of FHD, a biparental history of FHD, and a FHD, irrespective of the parent, were associated with T2DM onset. After adjustment for age, gender, BMI, and WHR, the consumption of potatoes, beef and mutton, fresh fruits, freshly squeezed juice, and soy products, and days of walking/week were associated with T2DM onset. There were significant interactions between FHD and consuming < 15 g/d of potatoes, <8 g/d of poultry, <85 g/d of fresh fruits, and no freshly squeezed juice.

This was a comprehensive investigation of the associations between dietary habits and T2DM in the Chinese. Potatoes have hypoglycemic activity in diabetic patients [25], and the present study showed an association between consuming < 15 g/d of potatoes and T2DM. High intake of red meat such as beef and mutton has been associated with T2DM [26], supporting the present study, that is, that consuming > 4 g/d of beef and mutton was associated with T2DM. The present study also showed that consuming < 85 g/d of fresh fruits and no freshly squeezed fruit and vegetable juices was associated with T2DM, which is supported by two European prospective studies [27, 28]. Although some clinical studies supported the antidiabetic effects of vegetables and soy products in Asians [29–31], the present study suggested that consuming > 30 g/d of soy products was associated with T2DM. This discrepancy may be explained by the facts that soy products are often cooked with red meat and drinking sweetened soybean milk in middle China.

This is the first study examining the interaction between dietary factors and FHD on T2DM onset. There were significant interactions between FHD and consuming < 15 g/d of potatoes, <8 g/d of poultry, <85 g/d of fresh fruits, and no freshly squeezed juice. Of course, the individual dietary habits are influenced by the familial dietary habits (PMID: 27050725) [32], and the present study could not tell the amplitude of this influence in relation to FHD. Nevertheless, the present study provides clues about possible changes in dietary habits in individual without T2DM but with FHD.

It was observed that patients with FHD had earlier age at onset of T2DM than those without, indicating that FHD might lead to earlier occurrence of the disease, suggesting genetic and environmental (family) influences on T2DM onset [12]. The proportion of female with FHD was higher than male in both T2DM and non-T2DM groups, which suggested positive FHD might have more influence for female who suffer from T2DM. In the present study, a biparental FHD was more strongly associated with the risk of T2DM, in agreement with earlier observations (in men and women combined) [15]. A higher risk of diabetes was observed in subjects with maternal history when compared with paternal history of diabetes. Previous study indicated that a stronger influence conferred by the mother compared to the father could be due to a larger contribution of diet, lifestyle factors, and adiposity from the mother [33].

The number of risk factors of T2DM in the FHD+ group is less than that of the FHD− group after adjustment for age and sex, which is similar to previous investigations [34–36]. Some relatively hidden factors such as passive smoking might increase the risk of suffering type 2 diabetes for the FHD+ population. In addition, some common risk factors for T2DM such as smoking, drinking, and obesity might increase the risk of T2DM for people without FHD. Previous smoking but not current smoking was associated with T2DM, which suggested patients may quit smoking after diagnosis of T2DM. This study also indicated more walking and higher BMI in T2DM group. The patients may increase physical activity deliberately but not enough to lose weight. The value of health education should be noticed.

Nevertheless, some limitations of this study should also be noted. Most of the information was obtained through an interview, resulting in possible inaccuracy in the risk factor metrics. The potential for recall bias was the second limitation. In studies with a cross-sectional design, a common limitation is the potential reverse causation bias. Associations between some risk factors and T2DM were in unexpected directions, and it might be due to an uncertainty whether exposure preceded the outcome or not. On the other hand, the subjects in the control group could have parents of diabetics, so the exposure level of control group might be raised, which would generate bias in the analysis of infected factors associated T2DM. The last issue might be information bias. A lot of measures were taken to minimize the biases. For example, our questionnaires were administered and checked by well-trained interviewers to exclude inter-interviewer variation, and we conducted the questionnaire study before the diseases were identified.

## 5. Conclusions

FHD was significantly associated with the risk of diabetes. There was a clear positive interaction between daily intake of potatoes and FHD, while an antagonistic interaction was observed between freshly squeezed vegetables and juices and FHD. FHD could have an appreciable influence on risk factors for T2DM and support that Chinese individuals with FHD should improve their lifestyle before T2DM onset.

## Figures and Tables

**Table 1:** Characteristics of the participants.

	T2DM	Non-T2DM	*P* value
	*N* (%)	*N* (%)	
Sex (male/female)	477/757	2705/5910	<0.001
Age (median, range)	63 (36, 90)	58 (23, 101)	<0.001
≤49	117 (9.5%)	1929 (22.4%)	<0.001
50–59	336 (27.3%)	2973 (34.5%)	—
60–69	481 (39.0%)	2508 (29.1%)	—
70–79	268 (21.8%)	1066 (12.4%)	—
≥80	30 (2.4%)	138 (1.6%)	—
BMI (median, range)	26.2 (15.8, 39.7)	25.7 (14.2, 39.6)	<0.001
<18.5	7 (0.6%)	77 (0.9%)	<0.001
18.5–23.9	283 (23.1%)	2534 (29.6%)	—
24–27.9	546 (44.6%)	3683 (43.0%)	—
≥28	388 (31.7%)	2282 (26.6%)	—
WHR (median, range)	0.918 (0.596, 1.600)	0.896 (0.420, 1.598)	<0.001

BMI: body mass index; WHR: waist-to-hip ratio.

**Table 2:** FHD and lifestyle of the participants.

	Variables	T2DM	Non-T2DM	*P* value
		*N* (%)	*N* (%)	
FHD	Yes	332 (26.9%)	1025 (11.9%)	<0.001
	No	902 (73.1%)	7590 (88.1%)	

Current smoking	Never	1063 (86.2%)	7278 (84.6%)	0.079
	<7 cigarettes/week	47 (3.8%)	285 (3.31%)	
	≥7 cigarettes/week	123 (10.0%)	1037 (12.1%)	

Previous smoking				<0.001
	Never	920 (81.9%)	6647 (86.3%)	<0.001
	<7 cigarettes/week	81 (7.2%)	496 (6.4%)	0.332
	≥7 cigarettes/week	123 (10.9%)	558 (7.3%)	<0.001

Current drinking	Never	988 (80.1%)	6719 (78.1%)	0.209
	<once/week	171 (13.9%)	1264 (14.7%)	
	≥once/week	74 (6.0%)	616 (7.2%)	

Grain (g/d)	<300	249 (20.2%)	1682 (19.6%)	0.603
	≥300	984 (79.8%)	6915 (80.4%)	

Potatoes (g/d)	<15	761 (61.7%)	4328 (50.4%)	<0.001
	≥15	472 (38.3%)	4263 (49.6%)	

Pork (g/d)	<15	759 (61.6%)	4856 (56.5%)	<0.001
	≥15	473 (38.4%)	3735 (43.5%)	

Beef and mutton (g/d)	<4	626 (50.8%)	4341 (50.5%)	0.874
	≥4	607 (49.2%)	4250 (49.5%)	

Poultry (g/d)	<8	713 (57.8%)	4568 (53.2%)	0.002
	≥8	520 (42.2%)	4024 (46.8%)	

Fish and seafood (g/d)	<7	724 (58.7%)	4943 (57.5%)	0.430
	≥7	509 (41.3%)	3649 (42.5%)	

Vegetable (g/d)	<300	452 (36.7%)	3081 (35.9%)	0.578
	≥300	781 (63.3%)	5514 (64.2%)	

Fresh fruits (g/d)	<85	834 (67.6%)	4126 (48.0%)	<0.001
	≥85	399 (32.4%)	4466 (52.0%)	

Freshly squeezed vegetable and fruit juices	Yes	181 (14.7%)	2301 (26.8%)	<0.001
	No	1052 (85.3%)	6288 (73.2%)	

Eggs (g/d)	<50	491 (39.8%)	3611 (42.0%)	0.137
	≥50	743 (60.2%)	4982 (58.0%)	

Soy products (g/d)	<30	779 (63.2%)	5569 (64.8%)	0.265
	≥30	454 (36.8%)	3025 (35.2%)	

Days of walking/week	≤3	310 (25.1%)	2748 (31.9%)	<0.001
	>3	924 (74.9%)	5867 (68.1%)	

Sitting time on weekdays (h/d)	<3	290 (23.5%)	2116 (24.6%)	0.329
	3–5	503 (40.8%)	3603 (41.8%)	
	>5	441 (35.7%)	2896 (33.6%)	

Sitting time on weekend (h/d)	<3	323 (26.2%)	2357 (27.4%)	0.674
	3–5	501 (40.6%)	3424 (39.7%)	
	>5	410 (33.2%)	2834 (32.9%)	

FHD: family history of diabetes.

**Table 3:** Associations of T2DM with a parental history of T2DM.

	Crude OR	95% CI	*P* value	Adjusted OR^∗^	95% CI	*P* value
Uniparental FHD	2.022	(1.698, 2.408)	<0.001	2.841	(2.358, 3.423)	<0.001
Paternal FHD	1.851	(1.416, 2.421)	<0.001	2.530	(1.911, 3.349)	<0.001
Maternal FHD	2.319	(1.913, 2.812)	<0.001	3.274	(2.669, 4.016)	<0.001
Biparental FHD	3.53	(2.029, 6.143)	<0.001	5.264	(2.975, 9.314)	<0.001
FHD	2.726	(2.366, 3.141)	<0.001	3.586	(3.082, 4.173)	<0.001

^∗^Adjusted OR for age, gender, body mass index, and waist-to-hip ratio. FHD: family history of diabetes.

**Table 4:** Multivariate analysis of associations between T2DM and lifestyle.

Variables		*N*	Crude OR	95% CI	*P*	*N*	Adjusted OR^∗^	95% CI	*P*
Age							1.045	(1.038, 1.053)	<0.001

BMI							1.022	(1.003, 1.042)	0.021

WHR	Unit = 0.1						1.554	(1.386, 1.742)	<0.001

FHD	Yes versus no						3.509	(2.999, 4.107)	<0.001

Sex	Male versus female						1.381	(1.165, 1.637)	<0.001

Current smoking	Never	8305	—	—	—	8254	—	—	—
	<7 cigarettes/week	330	1.28	(0.912, 1.794)	0.153	327	1.026	(0.719, 1.463)	0.888
	≥7 cigarettes/week	1156	0.831	(0.661, 1.045)	0.114	1150	0.734	(0.574, 0.939)	0.014

Current drinking	Never	7679	—	—	—	7630	—	—	—
	<once/week	1426	0.889	(0.734, 1.078)	0.232	1418	0.785	(0.636, 0.968)	0.024
	≥once/week	686	0.798	(0.601, 1.061)	0.12	683	0.672	(0.498, 0.906)	0.009

Grain (g/d)	≥300	7877	—	—	—	7832	—	—	—
	<300	1914	1.001	(0.852, 1.175)	0.993	1899	1.04	(0.879, 1.229)	0.649

Potatoes (g/d)	≥15	4720	—	—	—	4692	—	—	—
	<15	5071	1.411	(1.236, 1.61)	<0.001	5039	1.489	(1.297, 1.709)	<0.001

Pork (g/d)	≥15	4199	—	—	—	4175	—	—	—
	<15	5592	1.121	(0.978, 1.284)	0.1	5556	1.051	(0.911, 1.212)	0.495

Beef and mutton (g/d)	≥4	4836	—	—	—	4801	—	—	—
	<4	4955	0.768	(0.667, 0.884)	<0.001	4930	0.782	(0.675, 0.907)	0.001

Poultry (g/d)	≥8	4529	—	—	—	4503	—	—	—
	<8	5262	1.116	(0.968, 1.287)	0.13	5228	1.1	(0.947, 1.277)	0.211

Fish and seafood (g/d)	≥7	4144	—	—	—	4120	—	—	—
	<7	5647	0.876	(0.762, 1.008)	0.065	5611	0.847	(0.731,0.98)	0.026

Vegetable (g/d)	≥300	6274	—	—	—	6236	—	—	—
	<300	3517	0.973	(0.849, 1.114)	0.687	3495	0.921	(0.8, 1.061)	0.255

Fresh fruits (g/d)	≥85	4846	—	—	—	4818	—	—	—
	<85	4945	2.483	(2.175, 2.835)	<0.001	4913	2.339	(2.038, 2.684)	<0.001

Freshly squeezed vegetable and fruit juices	Yes	2478	—	—	—	2464	—	—	—
	No	7313	2.429	(2.035, 2.899)	<0.001	7267	2.232	(1.86, 2.679)	<0.001

Egg (g/d)	<50	3380	—	—	—	3358	—	—	—
	≥50	706	1.185	(1.043, 1.346)	0.009	700	1.122	(0.981, 1.283)	0.092

Soy products (g/d)	<30	5705	—	—	—	5673	—	—	—
	≥30	6325	1.244	(1.089, 1.42)	0.001	6282	1.191	(1.037, 1.368)	0.013

Days of walking/week	≤3	1510	—	—	—	1506	—	—	—
	>3	1956	1.418	(1.231, 1.634)	<0.001	1943	1.29	(1.114, 1.494)	0.001

Sitting time on weekdays (h/d)	<3	3040	—	—	—	3022	—	—	—
	3–5	6751	0.981	(0.781, 1.232)	0.869	6709	0.99	(0.779, 1.258)	0.934
	>5	2380	1.367	(1.018, 1.836)	0.038	2363	1.385	(1.015, 1.888)	0.04

Sitting time on weekend (h/d)	<3	4086	—	—	—	4069	—	—	—
	3–5	3325	1.046	(0.838, 1.305)	0.692	3299	0.986	(0.781, 1.245)	0.905
	>5	2650	0.826	(0.618, 1.104)	0.197	2638	0.747	(0.551, 1.014)	0.061

^∗^Adjusted OR for age, gender, body mass index, waist-to-hip ratio, and family history of diabetes.

**Table 5:** Interaction effects between FHD and dietary factors.

Exposure	Number of exposed cases	OR of diabetes	*S* (95% CI)
Grain ≥ 300 without FHD (reference)	6818		
Grain < 300 without FHD	1659	1.061 (0.894, 1.26)	
Grain ≥ 300 with FHD	1081	2.791 (2.382, 3.27)	
Grain < 300 with FHD	272	2.672 (2.001, 3.568)	0.902 (0.547, 1.489)
Pork ≥ 15 without FHD (reference)	3560		
Pork < 15 without FHD	4912	1.306 (1.131, 1.507)	
Pork ≥ 15 with FHD	648	2.876 (2.316, 3.571)	
Pork < 15 with FHD	703	3.557 (2.905, 4.356)	1.172 (0.83, 1.657)
Potatoes ≥ 15 without FHD (reference)	4066		
Potatoes < 15 without FHD	4405	1.599 (1.388, 1.843)	
Potatoes ≥ 15 with FHD	669	2.694 (2.161, 3.358)	
Potatoes < 15 with FHD	684	4.529 (3.713, 5.525)	1.539 (1.117, 2.121)
Beef and mutton ≥ 4 without FHD (reference)	4132		
Beef and mutton < 4 without FHD	4339	1.002 (0.873, 1.151)	
Beef and mutton ≥ 4 with FHD	725	2.537 (2.08, 3.096)	
Beef and mutton < 4 with FHD	628	2.974 (2.426, 3.645)	1.282 (0.856, 1.919)
Poultry ≥ 8 without FHD (reference)	3841		
Poultry < 8 without FHD	4631	1.215 (1.056, 1.398)	
Poultry ≥ 8 with FHD	703	2.545 (2.063, 3.139)	
Poultry < 8 with FHD	650	3.649 (2.981, 4.465)	1.505 (1.044, 2.171)
Fish and seafood ≥ 7 without FHD (reference)	3546		
Fish and seafood < 7 without FHD	4926	1.048 (0.91, 1.206)	
Fish and seafood ≥ 7 with FHD	612	2.585 (2.081, 3.212)	
Fish and seafood < 7 with FHD	741	2.999 (2.463, 3.652)	1.224 (0.821, 1.824)
Vegetable ≥ 300 without FHD (reference)	5400		
Vegetable < 300 without FHD	3075	1.055 (0.915, 1.217)	
Vegetable ≥ 300 with FHD	895	2.744 (2.302, 3.273)	
Vegetable < 300 with FHD	458	2.875 (2.288, 3.613)	1.042 (0.693, 1.566)
Fresh fruits ≥ 85 without FHD (reference)	4166		
Fresh fruits < 85 without FHD	4307	2.222 (1.918, 2.574)	
Fresh fruits ≥ 85 with FHD	699	2.505 (1.979, 3.172)	
Fresh fruits < 85 with FHD	653	6.91 (5.651, 8.451)	2.167 (1.633, 2.877)
Freshly squeezed juice (yes) without FHD (reference)	2210		
Freshly squeezed juice (no) without FHD	6259	1.922 (1.599, 2.31)	
Freshly squeezed juice (yes) with FHD	272	2.005 (1.349, 2.98)	
Freshly squeezed juice (no) with FHD	1081	5.342 (4.313, 6.616)	2.253 (1.456, 3.487)
Egg < 50 without FHD (reference)	3560		
Egg ≥ 50 without FHD	4915	1.102 (0.957, 1.268)	
Egg < 50 with FHD	1081	2.833 (2.263, 3.548)	
Egg ≥ 50 with FHD	810	2.934 (2.419, 3.559)	0.999 (0.681, 1.467)
Soy products < 30 without FHD (reference)	5506		
Soy products ≥ 30 without FHD	1659	1.03 (0.892, 1.19)	
Soy products < 30 with FHD	842	2.629 (2.194, 3.149)	
Soy products ≥ 30 with FHD	511	2.989 (2.409, 3.707)	1.199 (0.803, 1.79)

FHD: family history of diabetes.

## References

[1] Lozano R., Naghavi M., Foreman K. (2012). Global and regional mortality from 235 causes of death for 20 age groups in 1990 and 2010: a systematic analysis for the Global Burden of Disease Study 2010. *The Lancet*.

[2] Murray C. J. L., Vos T., Lozano R. (2012). Disability-adjusted life years (DALYs) for 291 diseases and injuries in 21 regions, 1990–2010: a systematic analysis for the Global Burden of Disease Study 2010. *The Lancet*.

[3] Yang W., Lu J., Weng J. (2010). Prevalence of diabetes among men and women in China. *New England Journal of Medicine*.

[4] van Dam R. M., Rimm E. B., Willett W. C., Stampfer M. J., Hu F. B. (2002). Dietary patterns and risk for type 2 diabetes mellitus in U.S. men. *Annals of Internal Medicine*.

[5] Fung T. T., Schulze M., Manson J. E., Willett W. C., Hu F. B. (2004). Dietary patterns, meat intake, and the risk of type 2 diabetes in women. *Archives of Internal Medicine*.

[6] Astrup A., Dyerberg J., Selleck M., Stender S. (2008). Nutrition transition and its relationship to the development of obesity and related chronic diseases. *Obesity Reviews*.

[7] Sun Z., Zheng L., Detrano R. (2010). Incidence and predictors of hypertension among rural Chinese adults: results from Liaoning province. *Annals of Family Medicine*.

[8] Zhang X., Sun Z., Zhang X. (2008). Prevalence and associated factors of overweight and obesity in a Chinese rural population. *Obesity (Silver Spring)*.

[9] Schmidt M. I., Duncan B. B., Bang H. (2005). Identifying individuals at high risk for diabetes: The atherosclerosis risk in communities study. *Diabetes Care*.

[10] Wikner C., Gigante B., Hellenius M. L., de Faire U., Leander K. (2013). The risk of type 2 diabetes in men is synergistically affected by parental history of diabetes and overweight. *PloS One*.

[11] Wilson P. F., Meigs J. B., Sullivan L., Fox C. S., Nathan D. M., D’Agostino R. B. (2007). Prediction of incident diabetes mellitus in middle-aged adults: the Framingham offspring study. *Archives of Internal Medicine*.

[12] InterAct Consortium (2013). The link between family history and risk of type 2 diabetes is not explained by anthropometric, lifestyle or genetic risk factors: the EPIC-InterAct study. *Diabetologia*.

[13] Carey V. J., Walters E. E., Colditz G. A. (1997). Body fat distribution and risk of non-insulin-dependent diabetes mellitus in women: the Nurses’ Health Study. *American Journal of Epidemiology*.

[14] Manson J. E., Stampfer M. J., Colditz G. A. (1991). Physical activity and incidence of non-insulin-dependent diabetes mellitus in women. *The Lancet*.

[15] Narayan K. M. V., Boyle J. P., Thompson T. J., Gregg E. W., Williamson D. F. (2007). Effect of BMI on lifetime risk for diabetes in the US. *Diabetes Care*.

[16] Van’t Riet E., Dekker J. M., Sun Q., Nijpels G., Hu F. B., van Dam R. M. (2010). Role of adiposity and lifestyle in the relationship between family history of diabetes and 20-year incidence of type 2 diabetes in U.S. women. *Diabetes Care*.

[17] Ning G., Reaction Study Group (2012). Risk evaluation of cAncers in Chinese diabeTic individuals: a lONgitudinal (REACTION) study. *Journal of Diabetes*.

[18] Bi Y., Lu J., Wang W. (2014). Cohort profile: risk evaluation of cancers in Chinese diabetic individuals: a longitudinal (REACTION) study. *Journal of Diabetes*.

[19] Luepker R. V., Evans A., McKeigue P., Reddy K. S. (2004). *Cardiovascular Survey Methods*.

[20] Andersson T., Alfredsson L., Källberg H., Zdravkovic S., Ahlbom A. (2005). Calculating measures of biological interaction. *European Journal of Epidemiology*.

[21] Kalilani L., Atashili J. (2006). Measuring additive interaction using odds ratios. *Epidemiologic Perspectives & Innovations*.

[22] Ahlqvist E., Ahluwalia T. S., Groop L. (2011). Genetics of type 2 diabetes. *Clinical Chemistry*.

[23] Shaw J. E., Sicree R. A., Zimmet P. Z. (2010). Global estimates of the prevalence of diabetes for 2010 and 2030. *Diabetes Research and Clinical Practice*.

[24] Gillies C. L., Abrams K. R., Lambert P. C. (2007). Pharmacological and lifestyle interventions to prevent or delay type 2 diabetes in people with impaired glucose tolerance: systematic review and meta-analysis. *BMJ*.

[25] Chandrasekara A., Josheph Kumar T. (2016). Roots and tuber crops as functional foods: a review on phytochemical constituents and their potential health benefits. *International Journal of Food Science*.

[26] Ekmekcioglu C., Wallner P., Kundi M., Weisz U., Haas W., Hutter H. P. (2016). Red meat, diseases and healthy alternatives: a critical review. *Critical Reviews in Food Science and Nutrition*.

[27] Heidemann C., Hoffmann K., Spranger J. (2005). A dietary pattern protective against type 2 diabetes in the European Prospective Investigation into Cancer and Nutrition (EPIC)–Potsdam study cohort. *Diabetologia*.

[28] Mursu J., Virtanen J. K., Tuomainen T. P., Nurmi T., Voutilainen S. (2014). Intake of fruit, berries, and vegetables and risk of type 2 diabetes in Finnish men: the Kuopio Ischaemic Heart Disease Risk Factor Study. *The American Journal of Clinical Nutrition*.

[29] Villegas R., Gao Y. T., Yang G. (2008). Legume and soy food intake and the incidence of type 2 diabetes in the Shanghai Women's Health Study. *The American Journal of Clinical Nutrition*.

[30] Mueller N. T., Odegaard A. O., Gross M. D. (2012). Soy intake and risk of type 2 diabetes in Chinese Singaporeans [corrected]. *European Journal of Nutrition*.

[31] Kwon D. Y., Daily J. W., Kim H. J., Park S. (2010). Antidiabetic effects of fermented soybean products on type 2 diabetes. *Nutrition Research*.

[32] Robson S. M., Couch S. C., Peugh J. L. (2016). Parent diet quality and energy intake are related to child diet quality and energy intake. *Journal of the Academy of Nutrition and Dietetics*.

[33] Abbasi A., Corpeleijn E., van der Schouw Y. T. (2011). Maternal and paternal transmission of type 2 diabetes: influence of diet, lifestyle and adiposity. *Journal of Internal Medicine*.

[34] Basu S., Stuckler D., McKee M., Galea G. (2013). Nutritional determinants of worldwide diabetes: an econometric study of food markets and diabetes prevalence in 173 countries. *Public Health Nutrition*.

[35] Bray G. A., Jablonski K. A., Fujimoto W. Y. (2008). Relation of central adiposity and body mass index to the development of diabetes in the diabetes prevention program. *The American Journal of Clinical Nutrition*.

[36] Sanz P. A., Boj C. D., Melchor L. I., Albero G. R. (2013). Sugar and diabetes: international recommendations. *Nutrición Hospitalaria*.

